# Nicotinamide Mononucleotide Administration Amends Protein Acetylome of Aged Mouse Liver

**DOI:** 10.3390/cells11101654

**Published:** 2022-05-16

**Authors:** Chengting Luo, Wenxi Ding, Songbiao Zhu, Yuling Chen, Xiaohui Liu, Haiteng Deng

**Affiliations:** 1 Tsinghua-Peking Center for Life Sciences, Tsinghua University, Beijing 100084, China; luoct@mail.tsinghua.edu.cn (C.L.); dwx18@mails.tsinghua.edu.cn (W.D.); zhusongbiao@mail.tsinghua.edu.cn (S.Z.); 2 MOE Key Laboratory of Bioinformatics, Center for Synthetic and Systematic Biology, School of Life Sciences, Tsinghua University, Beijing 100084, China; chenyuling2016@mail.tsinghua.edu.cn (Y.C.); xiaohui2013@mail.tsinghua.edu.cn (X.L.)

**Keywords:** acetylome, aging, nicotinamide mononucleotide, NAD (P) transhydrogenase, fatty acid β oxidation, TCA cycle

## Abstract

It is known that the activities of nicotine adenine dinucleotide (NAD^+^)-dependent deacetylase decline in the aging mouse liver, and nicotinamide mononucleotide (NMN)-mediated activation of deacetylase has been shown to increase healthspans. However, age-induced changes of the acetylomic landscape and effects of NMN treatment on protein acetylation have not been reported. Here, we performed immunoprecipitation coupled with label-free quantitative LC-MS/MS (IPMS) to identify the acetylome and investigate the effects of aging and NMN on liver protein acetylation. In total, 7773 acetylated peptides assigned to 1997 proteins were commonly identified from young and aged livers treated with vehicle or NMN. The major biological processes associated with proteins exhibiting increased acetylation from aged livers were oxidation-reduction and metabolic processes. Proteins with decreased acetylation from aged livers mostly participated in transport and translation processes. Furthermore, NMN treatment inhibited the aging-related increase of acetylation on proteins regulating fatty acid β oxidation, the tricarboxylic acid (TCA) cycle and valine degradation. In particular, NAD (P) transhydrogenase (NNT) was markedly hyperacetylated at K70 in aged livers, and NMN treatment decreased acetylation intensity without altering protein levels. Acetylation at cytochrome 3a25 (Cyp3a25) at K141 was also greatly increased in aged livers, and NMN treatment totally arrested this increase. Our extensive identification and analysis provide novel insight and potential targets to combat aging and aging-related functional decline.

## 1. Introduction

Post-translational modification of proteins has emerged as a highly conserved regulatory mechanism in eukaryotes. Protein acetylation, first discovered in 1963 [1], was found to modify many functions, including enzyme activities, DNA binding, subcellular location and protein stability [1]. Acetylation was found to regulate chromatin condensation and gene transcription by reversible histone acetylation [2,3]. Protein lysine residues can be acetylated with acetyl-CoA mediated by acetyltransferase or acetylated non-enzymatically [4,5]. In contrast, protein deacetylation needs a deacetylase, such as sirtuin proteins and histone deacetylases [4]. After a key observation that restriction of caloric intake can increase lifespan of mice and rats in 1939 [6], the elongation of healthspans and lifespans has gained growing interest from academic and medical researchers. Among longevity genes identified by genetic screening, yeast sirtuin 2 (*Sir2*) encodes a conserved protein that deacetylates the acyl group on histones [7,8]. Mice and humans express seven sirtuin proteins related to Sir2, including SIRT1–7 [9]. SIRT1, SIRT2, SIRT6 and SIRT7, which are mainly located in the nucleus and serve as epigenetic modulators [10]. SIRT3, SIRT4 and SIRT5 are located in mitochondria and regulate energy metabolic processes [11]. SIRT1 and SIRT3 are major deacetlases to regulate protein conformational changes and enzyme activities by removing acetyl modification of protein lysine residues [12,13]. Acetylation of proteins plays a vital role in regulating the aging process and age-related diseases. For example, mice with SIRT3 deficiency show a shorter lifespan than wild-type littermates [14]. Supplementation of nicotinamide mononucleotide (NMN) improved cytokine-mediated islet dysfunction mediated by SIRT1 [15]. Nicotinamide riboside (NR) attenuated oxidative stress and ameliorated liver functions via activation of SIRT1 and SIRT3 [16,17,18]. However, acetyl modifications of proteins during aging have been little explored.

Recent studies have reported mitigating age-induced physiological decline via NMN administration. NMN can be directly converted to nicotine adenine dinucleotide (NAD^+^) by nicotinamide/nicotinate mononucleotide adenylyltransferase [19]. NAD^+^ is central to energy metabolism as one of the most important cofactors for many biochemical reactions, as well as a substrate of NAD^+^-dependent enzymes, including SIRTs, poly adenosine diphosphate-ribose polymerase and the cyclic ADP-ribose synthase CD38/CD157 [20,21,22,23]. The level of NAD^+^ declines with aging in many cell types and tissues [23,24]. Supplementation of NAD^+^ precursors, such as NMN and NR, boosts the NAD^+^ pool and ameliorates age-related diseases [16,17,25,26]. For example, 12-month oral administration of NMN prevented age-induced bodyweight gain and improved hepatic insulin sensitivity, muscle mitochondrial biogenesis and energy expenditure [27]. NMN supplementation also ameliorated insulin resistance in age-induced diabetic mice [28]. Our previous studies also showed that the 4-week NMN administration alleviated CCL4-induced liver fibrosis and improved the function of peroxisomes in aged kidney [29]. Therefore, we aimed to investigate the effects of a short-term (4-week) NMN treatment on aged livers. Furthermore, studies suggested that NMN mitigated age-associated physiological decline via activation of SIRT1 and SIRT3 [27,30,31,32]. Therefore, it is possible that NMN administration markedly changed age-associated protein acetylation. However, the effects of NMN on protein acetylation during aging have not been investigated.

Here, we found that protein acetylation was globally increased in aged livers and NMN treatment arrested this increase. Then, we performed label-free quantitative acetylomic analysis using LC-MS/MS to identify specific acetylated sites. This study revealed that acetylation of many proteins was altered during aging and it comprehensively identified specific acetylated sites. These data also demonstrated that NMN treatment can amend aging-related acetylation alterations.

## 2. Materials and Methods

### 2.1. Animal Experiments

All animal experiments were conducted at the Laboratory Animal Research Center, Tsinghua University, with the approval of the Institutional Animal Care and Use Committee (IACUC) of Tsinghua University. C57BL/6 mice were purchased from Jackson Laboratory through Laboratory Animal Research Center, Tsinghua University. A total of 100 μL nicotinamide mononucleotide (NMN) solution (Sigma-Aldrich, 1094-61-7, dissolved in PBS, 500 mg/kg body weight) or PBS was intraperitoneally injected to 8-week- or 96-week-old mice every other day for 4 weeks, who were then sacrificed after 24 h of the last injection. Tissue was collected and cut into small pieces, frozen in liquid nitrogen immediately and stored at −80 °C.

### 2.2. Fractionation of Nuclear and Cytosolic Protein

Nuclear protein was extracted using a nucleus isolation kit according to manufacturer’s instructions (Solarbio, Beijing, China, R0050). Briefly, 30 mg liver was homogenized with a glass Dounce homogenizer in 500 μL lysis buffer, supplemented with protease inhibitor mixture (Solarbio, Beijing, China, P6730). Lysate was centrifugated at 700× *g* for 10 min at 4 °C. Protein concentration in supernatant solution was determined and boiled with protein loading buffer at 95 °C. The denatured protein sample containing cytosolic proteins was used to run western blot analysis. The pellet containing the nucleus was dissolved using lysis buffer and mixed with medium buffer and centrifuged at 700× *g* for 10 min at 4 °C. The supernatant was then discarded and the pellet resolved using lysis buffer. The protein concentration was determined and the nuclear sample denatured with protein loading buffer at 95 °C. 

### 2.3. Western Blot

A total of 50 mg tissue was homogenized with 500 μL RIPA buffer, containing protease inhibitor mixture (Solarbio, Beijing, China P6730). Supernatant was collected after centrifugating at 12,000 rpm at 4 °C for 30 min. Protein concentration was determined using a BCA kit (Thermo Fisher Scientific, Waltham, MA, USA, 23227). Protein was denatured by boiling with protein loading buffer at 95 °C for 10 min. An equal amount of protein was used to run 7.5% SDS gel to resolve proteins by electrophoresis, and then transferred to PVDF film. Antibody against acetylated lysine (Millipore, Burlington, MA, USA, 05-515), lamin B (Abcam, Waltham, MA, USA, ab16048) or β-actin (CST, Boston, MA, USA, 4970) was incubated overnight on a shaker at 4 °C. Films were washed with TBST 3 times and then incubated with second antibodies for 1 h at room temperature. Then, Image Lab software was used to visualize the signal under the mixture of ECLA and ECLB.

### 2.4. Acetylated Peptide Enrichment

Tissue was homogenized in lysis buffer (7 M urea, 2 M Thiourea, 50 mM Tris-HCl, PH 8.5, 3 μM TSA, 50 mM NAM, 50 mM sodium butyrate). Samples were then reduced with 5 mM TCEP and alkylated with 10 mM iodoacetamide for 1h at room temperature in the dark. Proteins were then precipitated with 4 vol of cold acetone at −20 °C for 4 h and collected by centrifugation at 4000 rpm at 4 °C for 10 min. Proteins were resuspended with 8 M urea in 10 mM HEPES (pH8.0) and protein concentration was determined using a BCA protein assay kit. Then, the protein solution was diluted fivefold by adding 50 mM NH_4_HCO_3_. Trypsin was added to the protein solution at a ratio of 1:50 trypsin to protein first for a 4 h digestion and a second digestion at a trypsin-to-protein ratio of 1:100 for another 12 h. Digested peptides were acidified with 50% trifluoroacetic acid (TFA) and desalted using 500 mg SEP PAK Classic C18 columns. Peptides were eluted with 50% acetonitrile in 0.5% acetic acid and stored at −80 °C overnight. To ensure the complete removal of TFA, peptide solutions were dried under the vacuum freezer dryer for another 48 h. Peptides with acetylated lysine were enriched using a PTMScan kit (Cell Signaling Technology, Boston, MA, USA, 13,416). Briefly, digested peptides were dissolved in 1.4 mL of 1 × IAP (immunoaffinity purification) buffer (50 mM MOPS pH 7.2, 10 mM sodium phosphate, 50 mM NaCl) and incubated with 80 μL prewashed beads, which were coupled with anti-aceK antibody at 4 °C for 2 h. Beads were washed three times with 1mL IAP buffer and three times with 1 mL of pre-chilled HPLC-grade water. Peptides were eluted from beads with 55 μL of 0.15% TFA two times. Eluted peptides were desalted using tips packed with 3 M Empore C18 disks. Peptides were dried under vacuum and resuspended in 10 μL 0.1% TFA for LC-MS/MS analysis.

### 2.5. Mass Spectrometry Analysis

The peptide solution was separated on a C18 reversed-phase column (75 μm inner-diameter, 150 mm length, 5 μm, 300 Å) using a Thermo-Dionex Ultimate 3000 HPLC system, which was coupled with a Q Exactive HF-X mass spectrometer (Thermo Fisher Scientific, Waltham, MA, USA). The HPLC solvent A was 0.1% FA and B was 80% acetonitrile with 0.08% FA. Peptides were eluted into the mass spectrometer at a flow rate of 300 nL/min. A linear gradient of 0.25% B/min with 240 min duration was used. The mass spectrometer was programmed to acquire in the data dependent acquisition mode. After one survey scan, the top 40 most intense peaks with charge state ≥ 2 were dissociated by a normalized collision energy of 27%. The scan range was set as 300–1800 m/z with a resolution of 60,000, AGC target of 3 × 10^6,^ and the maximum injection time was 120 ms. The MS2 spectra were acquired with a resolution of 15,000, isolation window of 1.6 m/z, AGC target of 1 × 10^5^ and maximum injection time (IT) of 50 ms.

### 2.6. Acetylated Peptides Searches

Mass spectral data sets were analyzed and searched using SEQUEST searching engine in Proteome Discoverer 2.3 software against the Uniprot Mouse database (2021_05). Search parameters for acetylated peptides were as follows: trypsin digestion with two missed cleavages was allowed. Trypsin specificity was set to C-terminal cleavage at lysine and arginine. Variable modifications included lysine acetylation and methionine oxidation. Carbamidomethyl cysteine was set as a fixed modification. Precursor ion and fragment ion mass tolerances were set to 20 ppm and 0.02 Da, respectively. The chromatographic peak area was used to quantitate the relative abundance of a peptide. Peptides with an expectation value <1% false discovery rate (FDR) were chosen for further data processing.

### 2.7. Quantitative Proteomic Analysis

Proteins were extracted from 20 mg liver homogenized in 500 μL RIPA buffer (20 mM Tris (pH7.5) 150 mM NaCl, 1% Triton X-100). Samples were centrifuged at 12,000× *g*, 15 min, and the supernatant was collected. Then, four times volume of iced acetone was added to precipitate protein at −20 °C for 4 h. All the liquid was removed by centrifuging at 2000× *g* for 20 min, then 8 M urea in PBS was added to each sample to dissolve the precipitate and measure protein concentration by BCA assay. A total of 100 μg protein from each sample was reduced with 5 mM dithiothreitol for 1 h at 37 °C, then alkylated with 12.5 mM iodoacetamide (IAM) in the dark at room temperature for 30 min. Proteins were then digested overnight at 37 °C with trypsin (Promega, Madison, WI, USA) at a ratio of 50:1 (protein: enzyme, *w*/*w*). Digested peptides were desalted using Sep-Pak desalting columns (Waters, Milford, MA 01757, USA) and peptide solutions were dried under a vacuum drier. The peptides were separately labeled with tandem mass tags (TMT) 6-plex reagents (Thermo Fisher Scientific, Waltham, MA, USA) according to manufacturer’s instructions. The labeled peptides were pooled and desalted by Sep-Pak columns. Labelled peptides were dried and dissolved in 300 μL water. Peptide solutions were fractioned into 48 fractions with reverse phase liquid chromatography using a Waters XBridgeTM BEH300 C18 column (Waters, Milford, MA, USA), and, lastly, were combined into 12 fractions. Peptides from the 12 fractions were dried and dissolved in 0.1% fumarate acid. Each peptide solution was subjected to LC-MS/MS analysis. The MS/MS spectra were analyzed with Proteome Discoverer 2.0 software and searched using the SEQUEST search engine. Search parameters in SEQUEST were as follows: trypsin digestion with at last two missed cleavages. Trypsin specificity was set to C-terminal cleavage at lysine and arginine. Methionine oxidation was set as a variable modification and carbamidomethylation at cysteine was set as a fixed modification. Precursor ion and fragment ion mass tolerances were set to 20 ppm and 0.02 Da, respectively. Peptides with <1% false discovery rate (FDR) were selected for further data processing.

### 2.8. Gene Ontology Analysis

Uniprot accession numbers of proteins with upregulated or downregulated acetylation were submitted to the DAVID bioinformatics functional annotation tool (http://david.abcc.ncifcrf.gov/, accessed on 15 February 2022) to identify enriched cellular components and biological processes (GOBP) terms. Mouse genes were chosen as the background population for all analyses.

### 2.9. Ingenuity Pathway Analysis

Uniprot accession numbers of proteins with upregulated or downregulated acetylation were uploaded to Ingenuity Pathway Analysis (IPA) software to perform pathway analysis. The pathways’ *p*-values were calculated based on Fisher’s exact test right-tailed methods. The match between observed gene expression and expected relationship direction is presented as a z-score. A Z-score > 2 is considered as a pathway that is significantly activated, and a z-score < −2 is considered as a pathway that is significantly inhibited.

### 2.10. Statistical Analysis

Data were expressed as mean ± standard deviation. Statistical analysis was performed using GraphPad Prism 7 software. Independent samples between two groups used student *t*-test to analyze differences of mean values and one-way ANOVA was used to evaluate differences among the mean values of more than two independent groups.

## 3. Results

### 3.1. Protein Acetylation Was Globally Enhanced and NMN Decreased This Acetylation in Aged Livers

**Figure 1:**
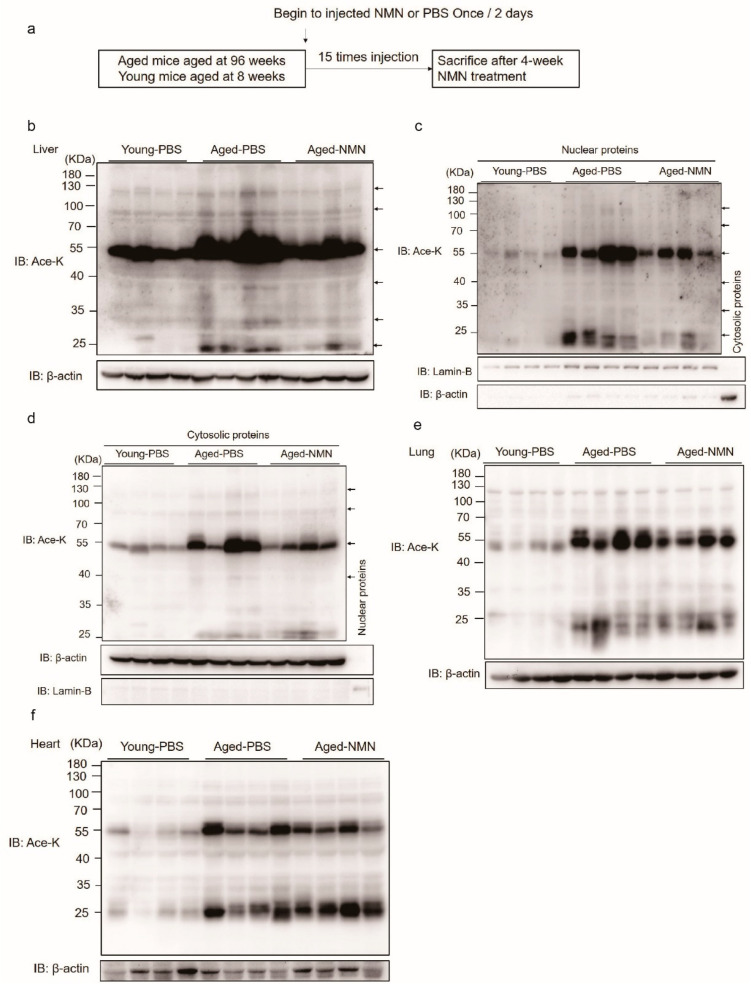
Protein acetylation in different organs and subcellular compartments in aged- and nicotinamide mononucleotide (NMN)-treated mice. (**a**) A graphical illustration of experiment procedure. (**b**–**f**) Western blot images of acetylated proteins from the liver (**b**), liver nuclear fraction (**c**), liver cytosolic fraction (**d**), lung and (**e**) heart, (**f**) in which an antibody against acetylated lysine was employed. Young-PBS: young mice treated with PBS, Aged-PBS: aged mice treated with PBS, Aged-NMN: aged mice treated with NMN.

To investigate the function and mechanism of NMN, NMN (500 mg/kg body weight) or an equal volume of PBS was intraperitoneally injected into aged mice every other day for 4 weeks (Figure 1a). Protein acetylation was dramatically enhanced in aged livers compared with young livers (Figure 1b and Supplementary Figure S3). NMN administration did not change the body weight of young and aged mice (Supplementary Figure S1), level of NAD^+^ and NADH or ratio of NAD^+^/NADH (Supplementary Figure S2), but NMN decreased the age-related acetylation of proteins (Figure 1b and Supplementary Figure S3). Furthermore, in aged livers, acetylation was enhanced on proteins located in the nucleus and cytosol, and NMN treatment decreased the acetylation intensity of both nuclear and cytosolic proteins (Figure 1c,d). Meanwhile, we examined the effects of NMN on liver protein acetylation in young mice by western blotting (Supplementary Figure S4), showing that NMN did not change global acetylation in young mice, and suggesting that the effects of NMN on liver protein acetylation were related to aging. In addition, heart and lung proteins were also dramatically acetylated in aged compared with young mice (Figure 1e,f). However, acetylation intensity of heart and lung proteins was not globally decreased by NMN treatment (Figure 1e,f). Interestingly, aged heart proteins of 25 kDa size were more acetylated after NMN treatment compared with PBS treatment (Figure 1f). These data suggested that global protein acetylation was related to hepatic aging, and NMN reduced the aging-associated liver protein acetylation.

### 3.2. Enrichment and Identification of the Liver Acetylome

**Figure 2:**
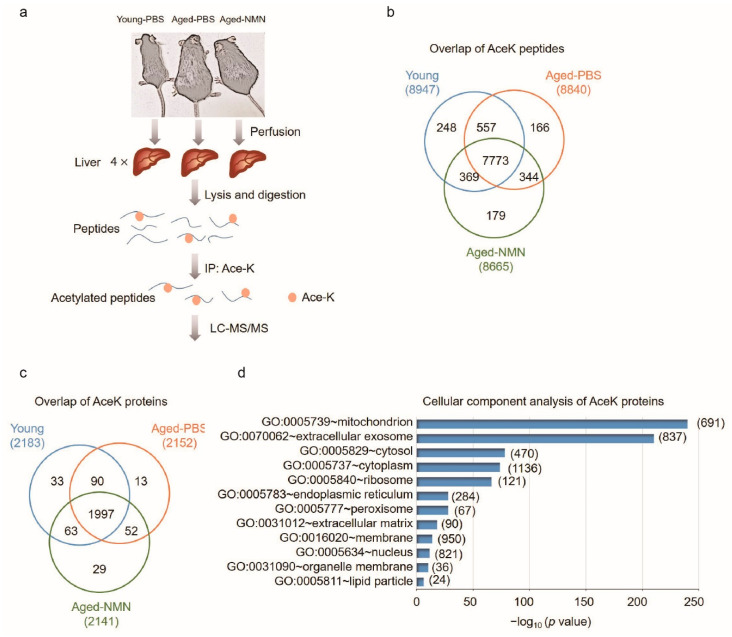
Identification of liver acetylome from young, untreated and NMN-treated aged mice. (**a**) A graphical illustration of liver acetylomic analysis using immunoprecipitation coupled with label-free quantitative LC-MS/MS (IPMS). (**b**) Numbers of acetylated peptides identified from young, aged-PBS and aged-NMN mouse liver. (**c**) Numbers of acetylated proteins identified from young, aged-PBS and aged-NMN mouse liver. (**d**) Cellular component analysis of the acetylated proteins that commonly identified from young, aged-PBS and aged-NMN mouse liver samples. Young-PBS: young mice treated with PBS, Aged-PBS: aged mice treated with PBS, Aged-NMN: aged mice treated with NMN.

To investigate the effects of aging and NMN on liver protein acetylation, we applied immunoprecipitation coupled with label-free quantitative LC-MS/MS (IPMS) to comprehensively identify the acetylome at lysine residues from young and aged mice treated with PBS (aged-PBS) and aged mice treated with NMN (aged-NMN) livers (Figure 2a). Mice were fed until tissue harvesting to minimize changes of SIRT1 and SIRT3 expression induced by fasting [33]. Each liver was perfused with ice-cold PBS to remove blood and avoid contamination with plasma proteins. Each liver was homogenized and an equal amount of protein was pooled for immunoprecipitation with an antibody against acetyl-lysine.

With a false discovery rate (FDR) cutoff ≤ 1%, the current analysis identified and quantified 8947, 8840 and 8665 unique peptides with lysine residue acetylation (AceK) from young, aged-PBS and aged-NMN livers (Figure 2b), respectively, resulting from 2183, 2152 and 2141 acetylated proteins, respectively (Figure 2c). In total, 7773 acetylated peptides across 1997 proteins were common to all three groups (Figure 2b,c). To determine whether a particular proteome subset was preferentially acetylated, we performed cellular component analysis of acetylated proteins overlapping across the young, aged-PBS and aged-NMN mouse livers (Figure 2d). Overall, 691 mitochondrial acetylated proteins, or nearly 40% of all mitochondrial proteins, were common to all three groups. This percentage of all mitochondrial acetylated proteins was higher than the 20% previously estimated [34]. A total of 950 proteins were identified as membrane proteins, and 837 as extracellular exosome proteins. In addition, 121 and 284 proteins were associated with the ribosome and endoplasmic reticulum, respectively. There were also 67 and 24 proteins associated with peroxisomes and lipid droplets, respectively. These data further extend the knowledge that post-translational modification (PTM) via acetylation is found on proteins associated with an extensive range of cellular organelles.

### 3.3. Aging-Induced Changes in the Acetylomic Landscape

**Figure 3:**
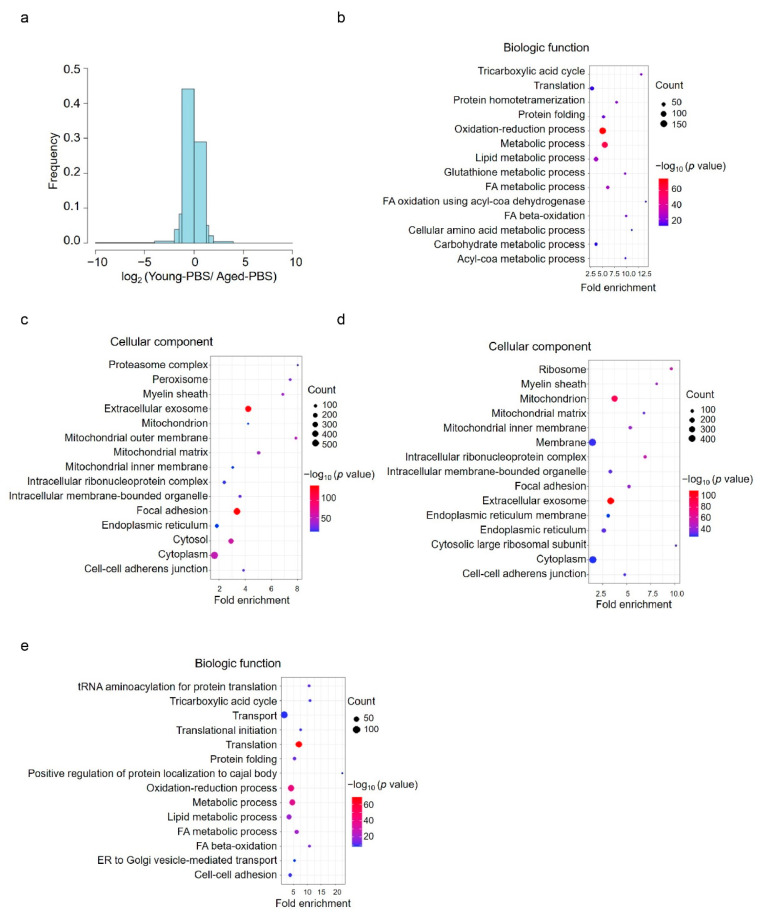
Analysis of acetylome in young and aged mice. (**a**) Frequency distribution showed more acetylated peptides were identified in aged mice than those in young mice. x axis was blocked at −10, −4, −2, −1.5, −1.25, 0, 1.25, 1.5, 2, 4, 10. (**b**) Biological function analysis of 997 acetylated proteins with increased acetylation in aged livers compared with young livers. (**c**) Cellular component distribution of 997 acetylated proteins with increased acetylation in aged livers compared with young livers. (**d**) Cellular component analysis of 845 acetylated proteins with reduced acetylation in aged livers compared with young livers. (**e**) Biological function analysis of 845 acetylated proteins with reduced acetylation in aged livers compared with young livers. Young-PBS: young mice treated with PBS, Aged-PBS: aged mice treated with PBS, Aged-NMN: aged mice treated with NMN.

To determine the effects of aging on lysine acetylation, we compared relative levels of acetylated peptides between young-PBS and aged-PBS samples using MS1 filtering label-free quantitation. Aging markedly increased the number of acetylated peptides (Figure 3a), consistent with western blotting findings (Figure 1a). Among the 7773 acetylated peptides quantified in all three groups, 2647 (34.1%) were acetylated more extensively in aged-PBS than in young liver samples (young:aged-PBS < 0.75, Supplementary Table S1). In contrast, 1679 (21.6%) peptides had relatively lower acetylation in the aged-PBS group than the young group (young:aged-PBS > 1.25, Supplementary Table S2). Gene ontology (GO) analysis showed that peptides with increased or decreased acetylation with aging had different cellular locations and exerted different biologic functions. Of the 997 proteins assigned to the 2647 peptides with increased acetylation, most were involved in oxidation-reduction and metabolic processes (Figure 3b), and subcellularly located in extracellular exosomes and focal adhesion sites (Figure 3c). However, of the 845 proteins associated with the 1679 peptides with reduced acetylation, most were classified as membrane proteins (Figure 3d) and related to transport and translation (Figure 3e).

**Figure 4:**
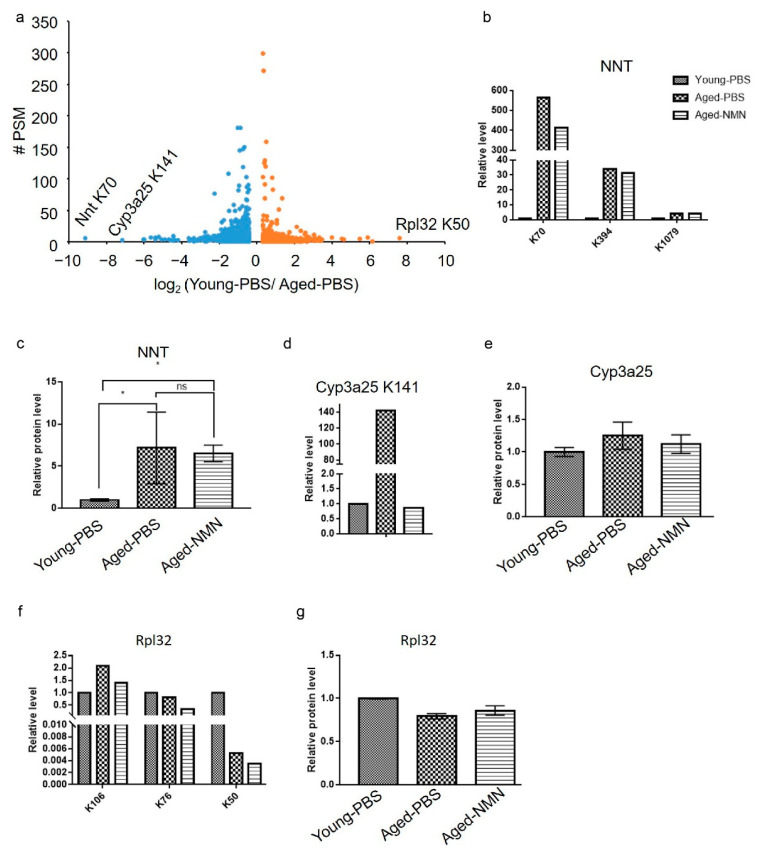
The fold changes distribution for acetylated peptides. (**a**) The volcano map showed that the acetylation level in NAD(P) transhydrogenase (NNT) at K70 was increased, whereas that in 60S ribosomal protein L32 (RPL32) at K50 was reduced in aged mice. PSM: peptide spectral matches. (**b**) The relative intensities of acetylated peptides from NNT in young, PBS-treated and NMN-treated aged mice. (**c**) The relative level of NNT protein quantitated with proteomic analysis. Data were expressed as mean ± SD, *n* = 4, * *p* < 0.05, ns: no significance. (**d**) The relative intensity of an acetylated peptide on Cyp3a25 in young, PBS-treated and NMN-treated aged mice. (**e**) The relative level of Cyp3a25 protein quantitated with proteomic analysis. Data were expressed as mean ± SD, *n* = 4. (**f**) The relative intensity of an acetylated peptide on RPL32 at K50 in young, PBS-treated and NMN-treated aged mice. (**g**) The relative level of Rpl32 protein quantitated with proteomic analysis. Data were expressed as mean ± SD, *n* = 4. Young-PBS: young mice treated with PBS, Aged-PBS: aged mice treated with PBS, Aged-NMN: aged mice treated with NMN.

Volcano analysis was performed to gain insight into the most affected acetylated peptides during the aging process. Interestingly, the largest increase in acetylation level was from protein NAD(P) transhydrogenase (NNT) at residue K70 (Figure 4a). Acetylation at two other sites on NNT, K394 and K1079, was also increased in aged compared with young livers (Figure 4b). NMN treatment counteracted the aging-related increase at K70 but not at K394 and K1079 (Figure 4b). The NNT protein level was increased approximately 6-fold in aged liver, and NMN treatment did not affect NNT expression (Figure 4c). Therefore, increased acetylation at K70 (nearly 600-fold), but not at K394 and K1079, was possibly related to the aging process, and NMN treatment contributed to deacetylation at K70. In contrast, increased acetylation at K394 and K1079 in the aged-PBS liver probably resulted from increased NNT protein levels, and NMN treatment had no effects on acetylation at K394 and K1079 and protein expression levels.

The second largest increased acetylation level was for a peptide from Cyp3a25 at K141 (Figure 4a), which has not been reported to date. Acetylation at Cyp3a25 K141 was dramatically increased in aged compared with young mice, and completely inhibited by NMN treatment (Figure 4d). Neither aging nor NMN treatment affected protein levels of Cyp3a25 (Figure 4e), suggesting acetylation at Cyp3a25 K141 participated in the regulation of the aging process and was potentially one of the targets of NMN.

In addition, the current study also identified acetylated peptides from 60S ribosomal protein L32 (RPL32) at K50, which exhibited the most reduced acetylation in aged-PBS compared with young samples (Figure 4a). Two other acetylated sites identified on RPL32, K106 and K76 showed similar acetylated levels among the three groups (Figure 4f). Protein expression levels of RPL32 were not changed by aging or NMN treatment (Figure 4g), suggesting that acetylation at K50 and the function of RPL32 were related to age, but NMN treatment had no effect on acetylation of RPL32.

### 3.4. NMN Changed Aging-Related Acetylome Involved in Key Metabolic Pathways

**Figure 5:**
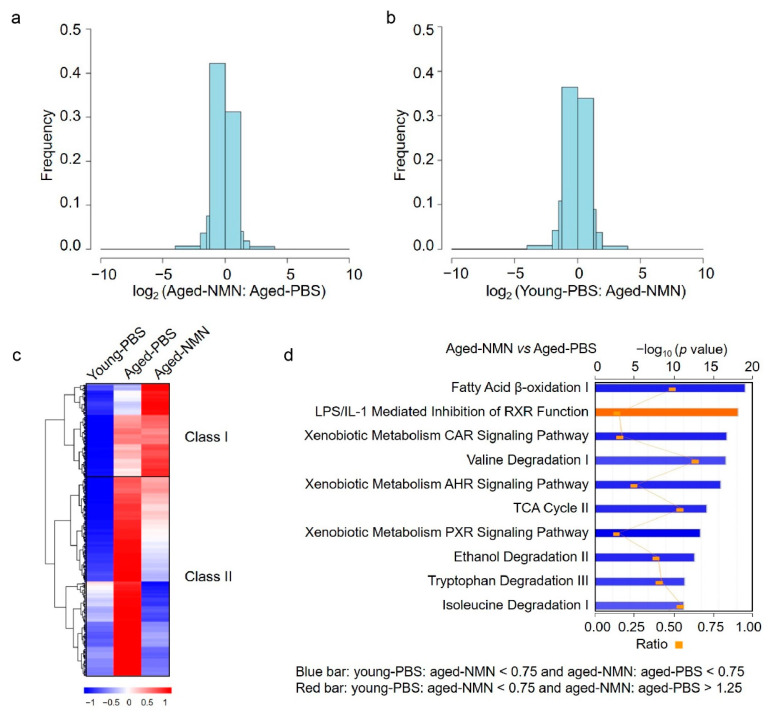
NMN changed aging-related acetylome. (**a**) Frequency distribution showed more acetylated peptides were identified in PBS-treated than NMN-treated aged mice. x axis was blocked at −10, −4, −2, −1.5, −1.25, 0, 1.25, 1.5, 2, 4, 10. (**b**) Frequency distribution showed similar numbers of acetylated peptides were identified in young and NMN-treated aged mice. x axis was blocked at −10, −4, −2, −1.5, −1.25, 0, 1.25, 1.5, 2, 4, 10. (**c**) NMN treatment decreased acetylation levels of most peptides of which acetylation was increased in aged PBS livers. (**d**) Ingenuity Pathway Analysis (IPA) of proteins with decreased or increased acetylation level by NMN treatment. Bars colored with blue or red indicated pathways enriched in proteins with decreased or increased acetylation level by NMN treatment, respectively. The red dots indicated number of proteins enriched in each pathway. Young-PBS: young mice treated with PBS, Aged-PBS: aged mice treated with PBS, Aged-NMN: aged mice treated with NMN.

**Figure 6:**
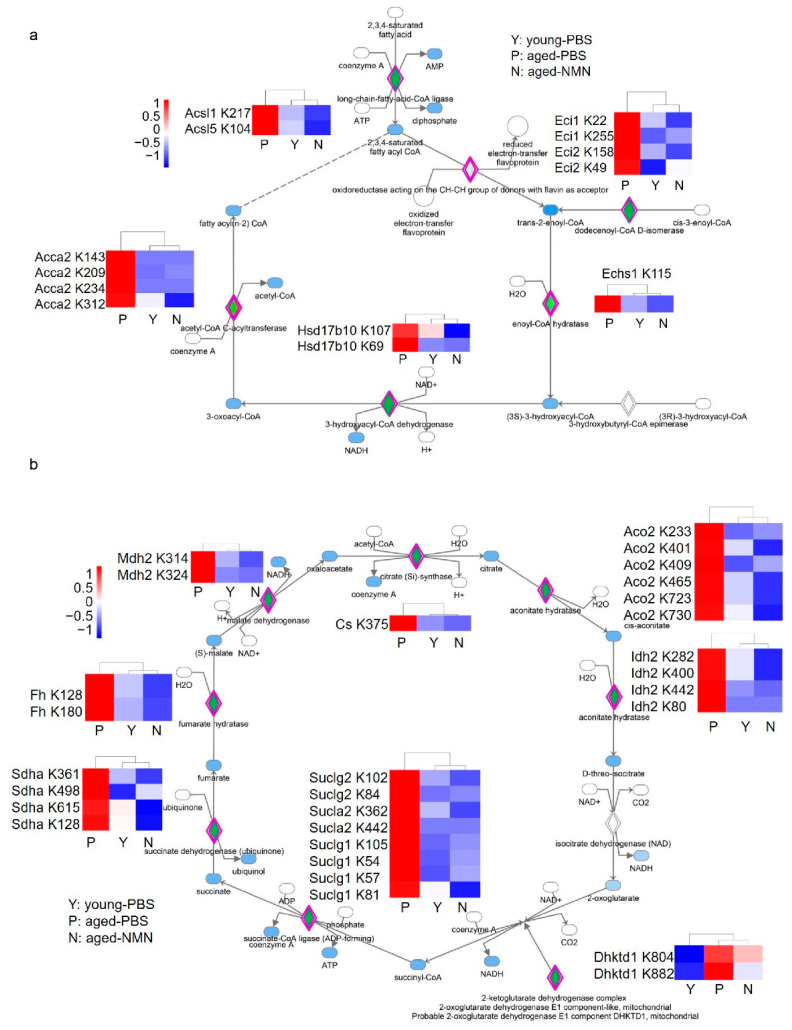
The effects of NMN on protein acetylation involved in fatty acid β oxidation and TCA cycle. (**a**) NMN treatment decreased aging-related enhancement of acetylation on enzymes catalyzing fatty acid β oxidation. Rhombuses colored with green indicated acetylated enzymes. Acaa2: mitochondrial 3-ketoacyl-CoA thiolase, Acsl1: long-chain-fatty-acid-CoA ligase 1, Acsl5: long-chain-fatty-acid-CoA ligase 5, Eci1: enoyl-CoA hydratase, Hsd17b10: 3-hydroxyacyl-CoA dehydrogenase type-2. (**b**) Enzymes participating in the TCA cycle were hyperacetylated in aged livers and NMN treatment reduced aging-related hyperacetylation. Cs: citrate synthase, Aco2: mitochondrial aconitate hydratase, Idh2: mitochondrial isocitrate dehydrogenase, Dhktd1: mitochondrial probable 2-oxoglutarate dehydrogenase E1 component, Suclg1: mitochondrial succinate-CoA ligase [ADP/GDP-forming] subunit alpha, Suclg2: mitochondrial succinate-CoA ligase [GDP-forming] subunit beta, Sucla2: mitochondrial succinate-CoA ligase [ADP-forming] subunit beta, Sdha: mitochondrial succinate dehydrogenase [ubiquinone] flavoprotein subunit, Fh: mitochondrial fumarate hydratase, Mdh2: mitochondrial malate dehydrogenase. Young-PBS: young mice treated with PBS, Aged-PBS: aged mice treated with PBS, Aged-NMN: aged mice treated with NMN.

To systematically evaluate the effects of NMN treatment on liver acetylomes, the relative intensities of acetylated peptides were compared between aged-PBS and aged-NMN livers. More peptides were hyperacetylated from aged-PBS than aged-NMN livers (Figure 5a), and the number of hyperacetylated peptides was similar between young and aged-NMN livers (Figure 5b). Quantitative proteomic analysis identified 5587 proteins from aged-PBS and aged-NMN liver samples and showed NMN treatment did not markedly affect protein expression levels (Supplementary Figure S5). These data were consistent with results from western blotting analysis (Figure 1a), demonstrating that aging causes more hyperacetylated peptides and NMN treatment arrested acetylation. Furthermore, NMN reduced the acetylation of most peptides (class II) of the 2647 peptides found to exhibit age-related acetylation in the liver (Figure 5c). Overall, 553 proteins were related to the 1171 class II peptides with acetylation levels markedly decreased by NMN supplementation (aged-NMN: aged-PBS < 0.75, Supplementary Table S3), and 266 proteins were assigned to the 356 class I peptides exhibiting increased acetylation after NMN treatment (aged-NMN: aged-PBS > 1.25, Supplementary Table S4). Ingenuity pathway analysis showed that most proteins exhibiting an aging-related increase in acetylation, which could also be reduced by NMN treatment, were involved in fatty acid β oxidation, xenobiotic metabolism, valine degradation and the TCA cycle (Figure 5d). For example, NMN treatment decreased the aging-related enhancement of acetylation on enzymes catalyzing fatty acid β oxidation, such as acetyl-CoA Acyltransferase 2, acyl-CoA synthetase long chain family member 1, acyl-CoA synthetase long chain family member 5, enoyl-CoA delta isomerase 1 and 17 beta-hydroxysteroid dehydrogenase type 10 (Figure 6a), whose expression levels were not changed by NMN treatment (Supplementary Figure S6a). Enzymes participating in the TCA cycle were also hyperacetylated in aged liver, and NMN treatment arrested age-related hyperacetylation (Figure 6b) without changing protein expression levels (Supplementary Figure S6b).

## 4. Discussion

The application of large-scale proteomic approaches found that a growing number of mitochondrial targets of acetylation play key roles in metabolism, such as acetyl-CoA synthetase, superoxide dismutase 2 and isocitrate dehydrogenase (IDH2) [34,35,36,37,38]. However, a large-scale investigation into the effects of aging and NMN treatment on protein acetylation has not been reported to date. The current study found that proteins were hyperacetylated during aging in tissues such as the liver, heart and lung, and NMN treatment markedly decreased acetylation on liver proteins in both the nucleus and the cytosol. Aging-induced protein hyperacetylation possibly resulted from declined NAD^+^ levels with aging in many organs, leading to dysfunction of NAD^+^-dependent deacetylase and hyperacetylation of target proteins [23]. In addition, this current study discovered that acetylation of enzymes participating in TCA were increased in aged livers compared with young livers, which could lead to inhibited enzymatic activities and consumption of acetyl-CoA via TCA in aged livers, and thus, it is possible that excess acetyl-CoA automatically modifies proteins. Moreover, acetylation on enzymes catalyzing fatty acid β oxidation was found increased in aged livers, and this provided a novel molecular mechanism to explain the observation that lipid was accumulated in aged livers [39]. NMN treatment reduced age-associated acetylation on TCA enzymes, indicating NMN may accelerate the usage of acetyl-CoA to decrease the potential of acetyl-CoA to modify proteins. Meanwhile, NMN treatment can activate SIRTs to deacetylate target proteins, supporting a previous observation that NMN activated SIRT1, SIRT3 and SIRT2 [27,31,40].

The largest increase in protein acetylation in aged compared with young livers was on NNT at K70 (nearly 600-fold), which was previously identified in the human acute myeloid leukemia cell line MV4-11 after treatment with a deacetylase inhibitor [41], suggesting acetylation at K70 was conserved. NMN treatment decreased the aging-related increase of K70 acetylation on NNT. The expression level of NNT protein was increased about 6-fold in aged livers and NMN treatment did not affect its expression level. NNT, assembled at the mitochondrial inner membrane, catalyzes the reduction of NADP^+^ at the expense of NADH oxidation, coupled with H^+^ flux to matrix from interspace [42]. A NNT loss-of-function mutation resulted in mitochondrial oxidative stress, dysfunction and impaired ATP production [43,44]. NNT has recently been found to consume NADH to maintain the balance between ATP synthesis and nutrient utilization, especially the oxidation of fatty acids [45,46]. NNT deficiency was reported to decrease HDAC1 activity and increase global protein acetylation in hepatoma cells [47]. We found that NNT proteins level was markedly increased in livers of aged mice, indicating that increased NNT level is one of factors to increase protein acetylation during aging. Meanwhile, NMN treatment decreased global protein acetylation without changing NNT protein levels, indicating NMN increased the activity of NNT by deacetylating at K70. Deacetylating at K70 did not change NNT protein levels, suggesting acetylation of NNT at K70 may regulate activity of NNT but not protein stability. In addition, a previous report showed that NNT affected IDH activity, TCA and ATP production via changing NADPH levels [47,48]. This study found that acetylation of TCA enzymes was increased and NADH production via TCA may be reduced in aged livers, suggesting reduced NNT activity in aged livers could be a feedback mechanism due to reduced NADH and NMN improved TCA and NNT activity. Therefore, acetylation at K70 probably plays a critical role in aging-related redox imbalance and mitochondrial dysfunction.

In addition, IDH2 is the other major dehydrogenase in mitochondria producing NADPH, the ultimate reducing source for H_2_O_2_ detoxification. Moreover, acetylation inhibits IDH2 homodimer formation and enzymatic activities, resulting in oxidative stress [49,50]. IDH2 produces NADPH to maintain redox homeostasis, and also provides intermediate metabolites for the TCA cycle to produce NADH, which is used to synthesize ATP through the electron transport chain. The current study found that acetylation of IDH2 was increased in aged liver and NMN treatment decreased this acetylation. This indicated that IDH2 may play an important role in coupling energy balance with redox homeostasis through reversible acetylation during aging.

## 5. Conclusions

All together, we found that protein acetylation was globally increased in aged livers and NMN treatment largely arrested this increase. Label-free quantitative acetylomics identified specific acetylated sites. NMN treatment inhibited the aging-related increase of acetylation on proteins regulating fatty acid β oxidation, the TCA cycle and valine degradation. In particular, hyperacetylation of NNT at K70 was observed in aged livers and NMN treatment decreased this acetylation without altering protein levels. This study has revealed that protein acetylation plays key roles in balancing redox homeostasis and energy metabolism during aging. NMN treatment can reprogram aging-related acetylation to reduce aging-associated dysfunctions.

## Data Availability

Data will be shared upon reasonable request.

## Supplementary Materials

The following are available online at https://www.mdpi.com/article/10.3390/cells11101654/s1. Figure S1: NMN administration did not change the body weight of young (a) and aged mice (b). Figure S2: NMN did not change NAD^+^ and NADH level or ratio of NAD^+^/NADH in aged (a–c) and young (d–e) mouse livers. Figure S3: Quantification of acetylated proteins from liver samples related to Figure 1b. Figure S4: Representative image of western blot of young liver proteins treated with an equal volume of PBS or NMN (intraperitoneally injected, 500 mg/kg body weight every other day for 4 weeks), probed against acetylated-lysine. Figure S5: NMN treatment did not dramatically affect protein expression level. Figure S6: Quantitative proteomic analysis showed that expression levels of enzymes catalyzing fatty acid β oxidation (a) and participating TCA cycle (b) were not changed by NMN treatment. Table S1: Peptides were relatively higher acetylated from aged-PBS livers than young livers. Table S2: Peptides were relatively lower acetylated from aged-PBS group than young group. Table S3: Among peptides with increased acetylation from aged-PBS livers compared with young livers, peptides were decreased by NMN supplementation. Table S4: Among peptides with increased acetylation from aged-PBS livers compared with young livers, peptides were increased by NMN treatment.
