# Supplementary material for: Nicotinamide Mononucleotide Administration Amends Protein Acetylome of Aged Mouse Liver
Source: Cells. 2022 May 16;11(10):1654. doi: 10.3390/cells11101654 (PMC9139684; doi:10.3390/cells11101654)
Supplement: Supplementary file 1 [file cells-11-01654-s001.zip › cells-1664524 -supplementary.pdf]

cells

MDPI

Supplementary Materials

Nicotinamide Mononucleotide Administration Amends Protein Acetylome of Aged Mouse Liver

Chengting Luo [1,2,†], Wenxi Ding [1,2,†], Songbiao Zhu [1,2], Yuling Chen [2], Xiaohui Liu [2] and Haiteng Deng [2,*]

[1] Tsinghua-Peking Center for Life Sciences, Tsinghua University, Beijing 100084, China; luoct@mail.tsinghua.edu.cn (C.L.); dwx18@mails.tsinghua.edu.cn (W.D.); zhusongbiao@mail.tsinghua.edu.cn (S.Z.)
[2] MOE Key Laboratory of Bioinformatics, Center for Synthetic and Systematic Biology, School of Life Sciences, Tsinghua University, Beijing 100084, China; chenyuling2016@mail.tsinghua.edu.cn (Y.C.); xiaohui2013@mail.tsinghua.edu.cn (X.L.)
* Correspondence: dht@mail.tsinghua.edu.cn
† These authors contributed equally to this work.

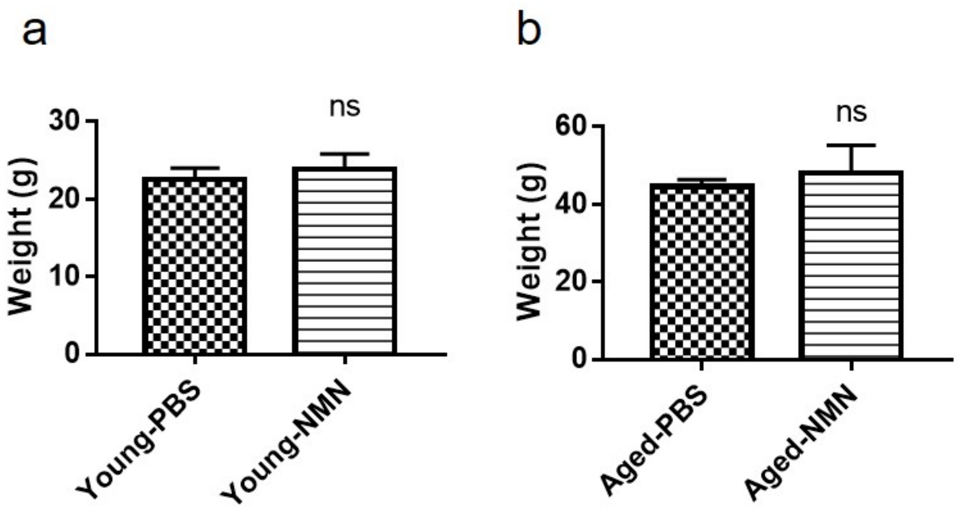

Figure S1. NMN administration did not change bodyweight of young (**a**) and aged mouse (**b**). Data were expressed as mean ± SD, $n = 4$.

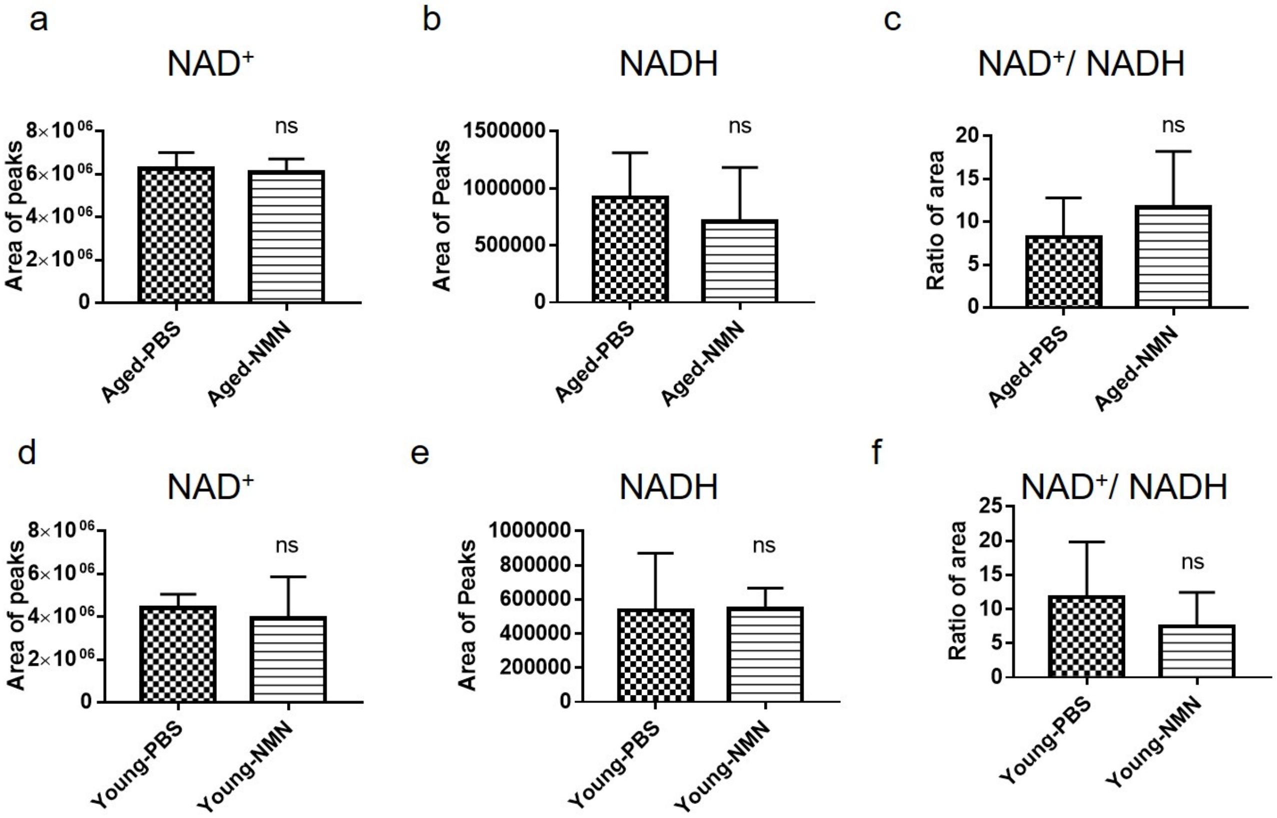

Figure S2. NMN did not change NAD+ and NADH level or ratio of NAD+/NADH in aged (**a**–**c**) and young (**d**–**e**) mouse livers. Data were expressed as mean ± SD, $n = 4$. Young-PBS: young mice treated with PBS, Aged-PBS: aged mice treated with PBS, Aged-NMN: aged mice treated with NMN.

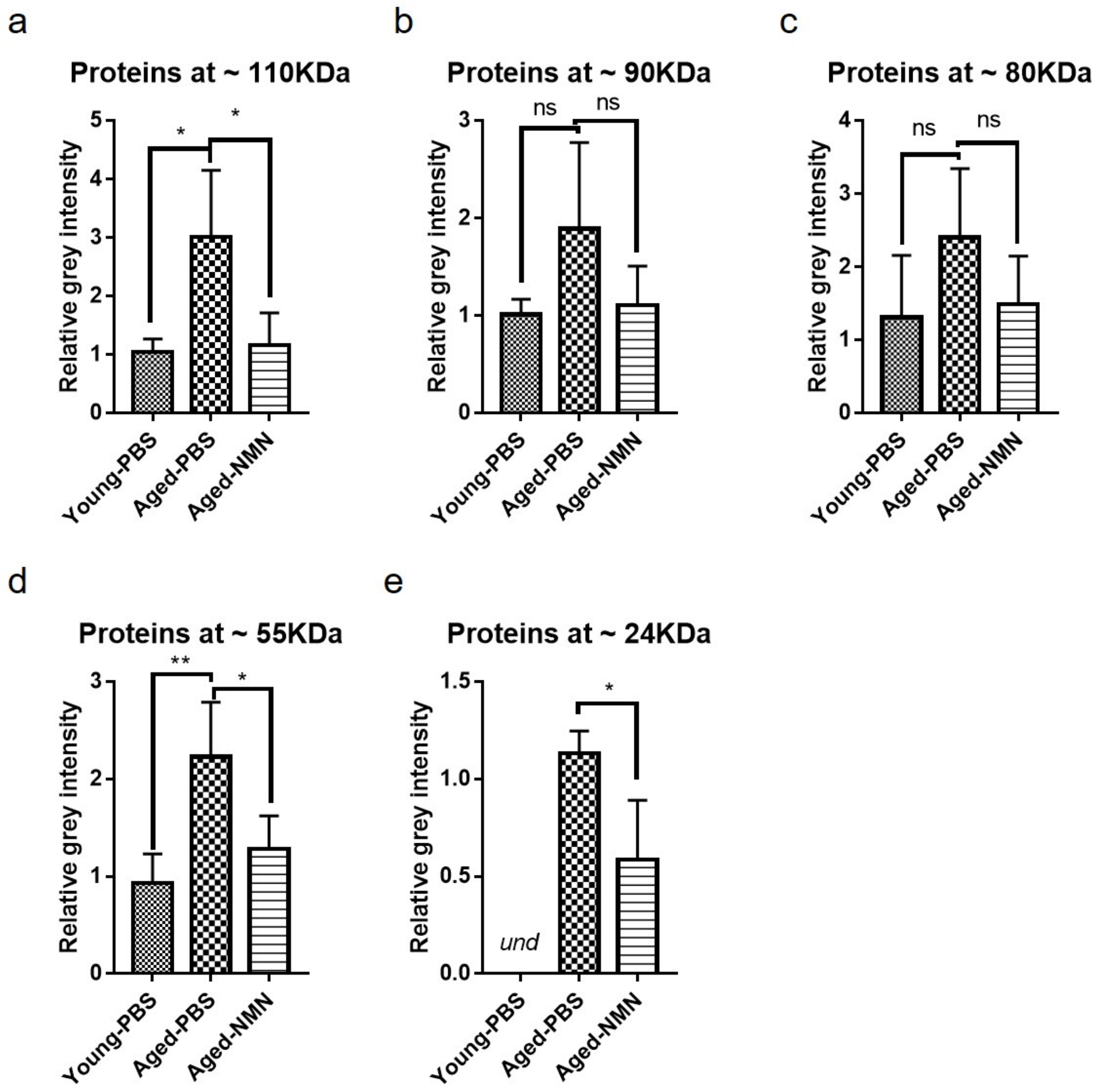

Figure S3. Quantification of acetylated proteins from liver sample related to Figure 1b. The intensity of selected bands was quantitated with Image Lab. Data were expressed as mean ± SD, $n = 4$.

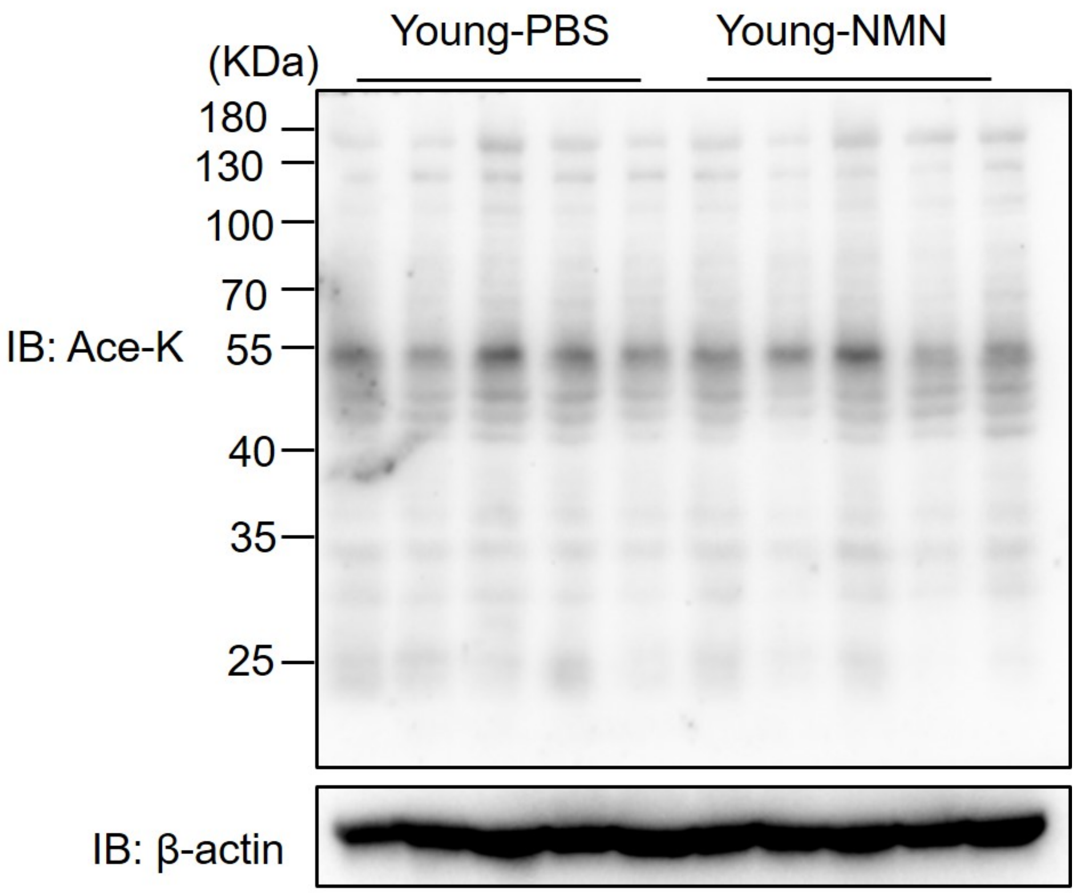

Figure S4. Representative image of western blot of young liver proteins treated with equal volume of PBS or NMN (intraperitoneally injected, 500 mg/kg body weight every other day for 4 weeks), probed against acetylated-lysine.

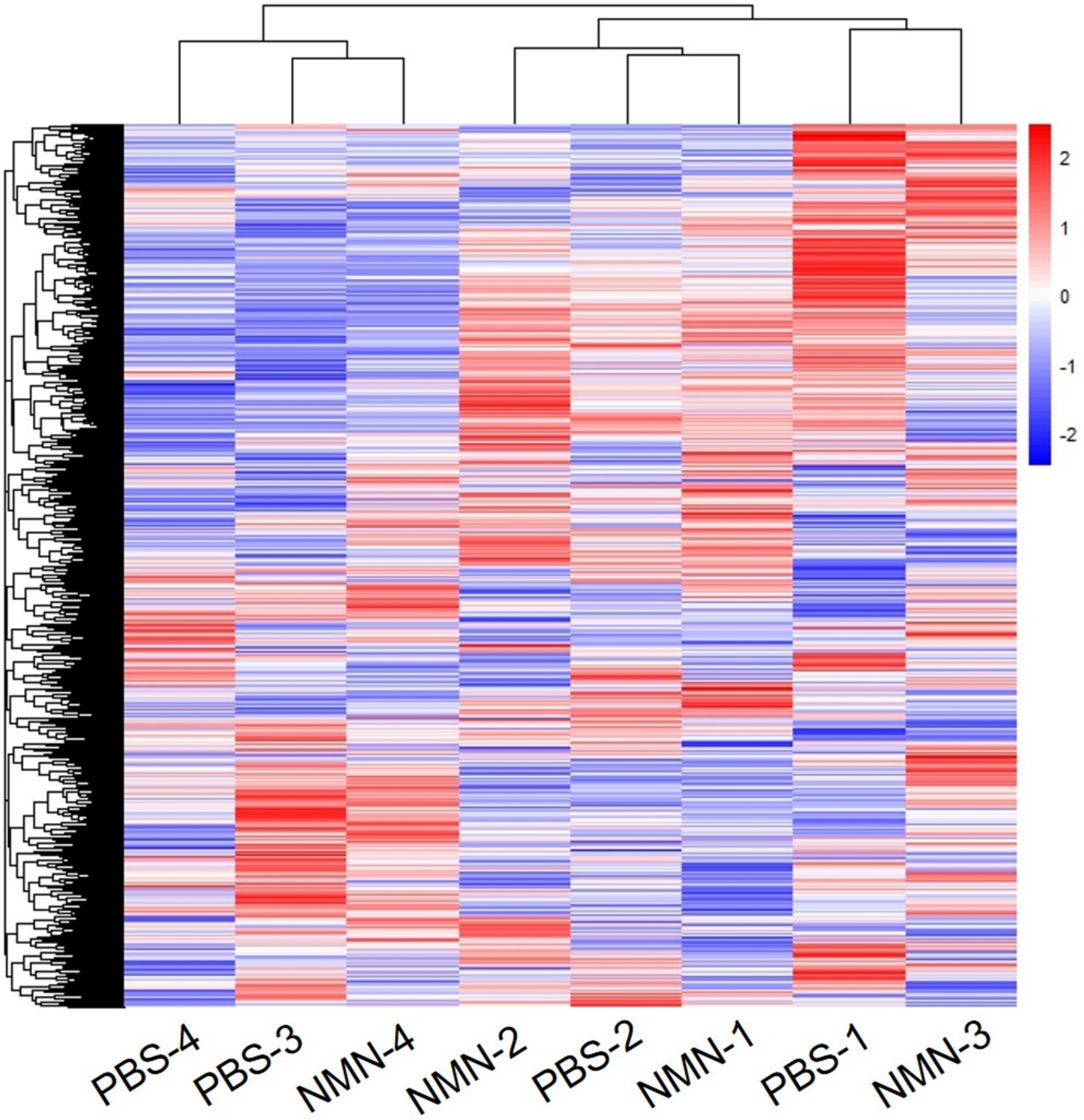

Figure S5. NMN treatment did not dramatically affect protein expression level. Quantitative proteomic analysis identified 5587 proteins from aged-PBS than aged-NMN liver and heat map analysis showed NMN treatment did not dramatically affect protein expression level. PBS: aged-PBS, NMN: aged-NMN, $n = 4$.

a

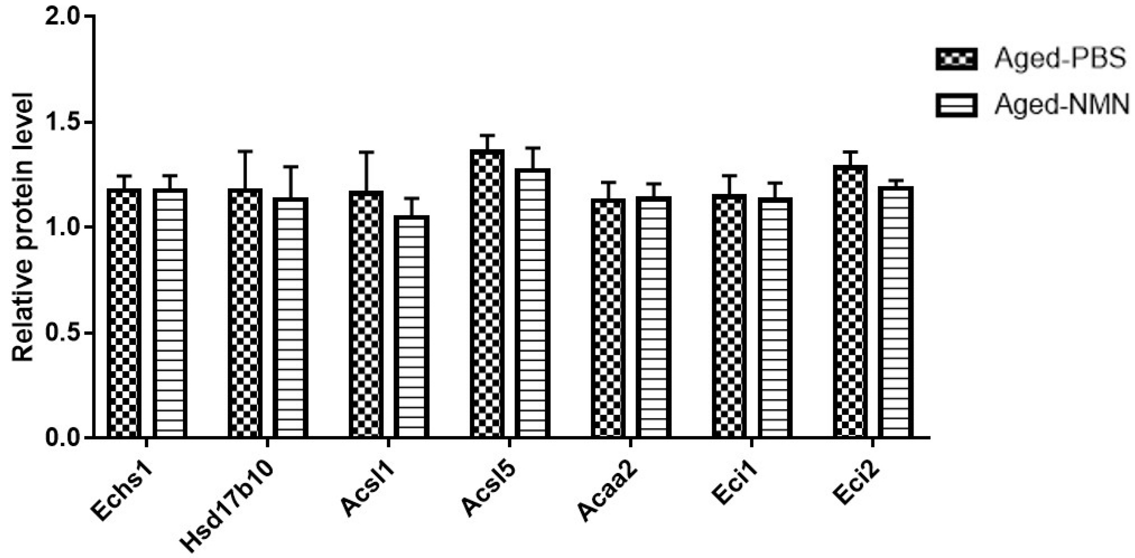

b

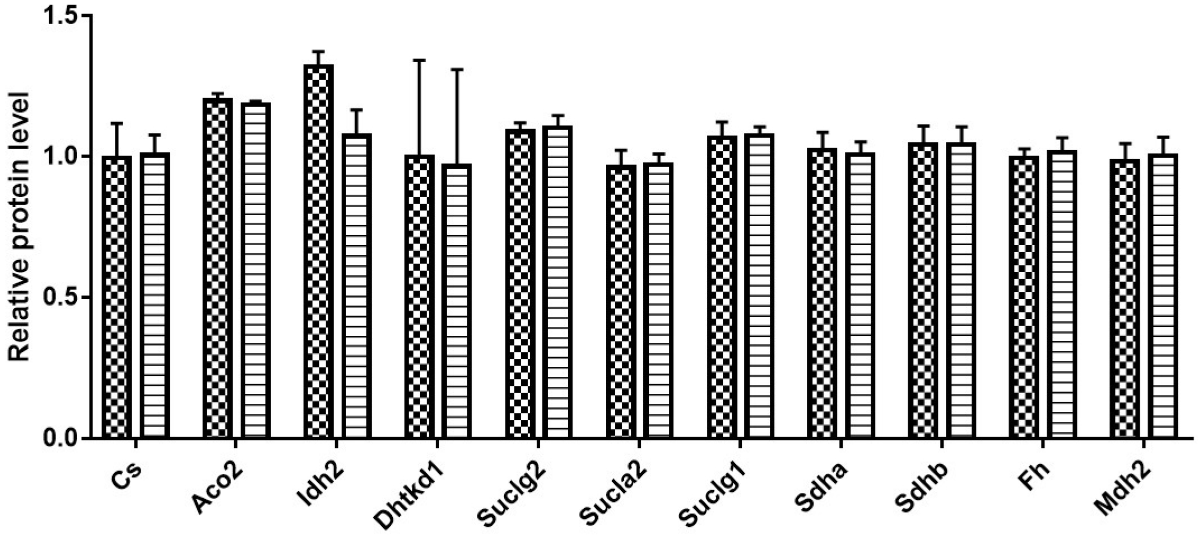

Figure S6. Quantitative proteomic analysis showed that expression levels of enzymes catalyzing fatty acid β oxidation (**a**) and participating TCA cycle (**b**) were not changed by NMN treatment. n = 4. Young-PBS: young mice treated with PBS, Aged-PBS: aged mice treated with PBS, Aged-NMN: aged mice treated with NMN.

Table S1. peptides were relatively higher acetylated from aged-PBS liver than young liver.

Table S2. peptides were relatively lower acetylated from aged-PBS group than young group.

Table S3. among peptides with increased acetylation from aged-PBS liver compared with young liver, peptides were decreased by NMN supplementation.

Table S4. among peptides with increased acetylation from aged-PBS liver compared with young liver, peptides were increased by NMN treatment.
